# Correction to: The RESIST Senior Individuals Cohort: Design, participant characteristics and aims

**DOI:** 10.1007/s11357-024-01380-0

**Published:** 2024-10-22

**Authors:** Lennart Matthias Roesner, Manoj Kumar Gupta, Verena Kopfnagel, Nienke van Unen, Yvonne Kemmling, Jana‑Kristin Heise, Stephanie Castell, Xun Jiang, Lennart Riemann, Stephan Traidl, Berit Lange, Kurt‑Wolfram Sühs, Thomas Illig, Till Strowig, Yang Li, Reinhold Förster, Jochen Huehn, Thomas Friedrich Schulz, Thomas Werfel, the RESIST SI Cohort Investigators

**Affiliations:** 1 Department of Dermatology and Allergy, Hannover Medical School (MHH), Hannover, Germany; 2 Cluster of Excellence RESIST (EXC 2155, Hannover Medical School (MHH), Hannover, Germany; 3 Hannover Unified Biobank (HUB), Hannover Medical School (MHH), Hannover, Germany; 4 Department of Computational Biology of Individualised Medicine, Centre for Individualised Infection Medicine (CiiM), a Joint Venture Between the Hannover Medical School, Helmholtz Centre for Infection Research, Hannover, Germany; 5 Department for Epidemiology, Helmholtz Centre for Infection Research (HZI), Braunschweig, Germany; 6 Institute of Immunology, Hannover Medical School (MHH), Hannover, Germany; 7 Department of Pediatric Pneumology, Allergology and Neonatology, Hannover Medical School, Hannover, Germany; 8 Department of Neurology, Hannover Medical School (MHH), Hannover, Germany; 9 Department of Microbial Immune Regulation, Helmholtz Centre for Infection Research (HZI), Braunschweig, Germany; 10 TWINCORE, Centre for Experimental and Clinical Infection Research, a Joint Venture Between the Hannover Medical School and the Helmholtz Centre for Infection Research, Hannover, Germany; 11 Department Experimental Immunology, Helmholtz Centre for Infection Research (HZI), Braunschweig, Germany; 12 Institute of Virology, Hannover Medical School (MHH), Hannover, Germany

**Correction to: GeroScience**

10.1007/s11357-024-01299-6

The original version of this article unfortunately contained errors in the affiliation and consortium sections.

The affiliations below were incorrectly captured as follows:

**Affiliation 3**

M. K. Gupta · V. Kopfnagel · T. Illig

Hannover Unified Biobank (HUB), Hannover Medical School (MHH), Hannover, Germany

**Affiliation 4**

N. van Unen · X. Jiang · Y. Li

Department of Computational Biology of Individualised Medicine, Centre for Individualised Infection Medicine (CiiM), a Joint Venture Between the Hannover Medical School and the Helmholtz Centre for Infection Research, Hannover, Germany

**Affiliation 10**

Y. Li

TWINCORE, Centre for Experimental and Clinical Infection Research, a Joint Venture Between the Hannover Medical School and the Helmholtz Centre for Infection Research, Hannover, Germany

The corrected affiliations are shown below.

**Affiliation 3**

V. Kopfnagel · T. Illig

Hannover Unified Biobank (HUB), Hannover Medical School (MHH), Hannover, Germany

**Affiliation 4**

M. K. Gupta · N. van Unen · X. Jiang · Y. Li

Department of Computational Biology of Individualised Medicine, Centre for Individualised Infection Medicine (CiiM), a Joint Venture Between the Hannover Medical School and the Helmholtz Centre for Infection Research, Hannover, Germany

**Affiliation 10**

M. K. Gupta · N. van Unen · X. Jiang · Y. Li

TWINCORE, Centre for Experimental and Clinical Infection Research, a Joint Venture Between the Hannover Medical School and the Helmholtz Centre for Infection Research, Hannover, Germany

The consortium section below was incorrectly captured as follows:

**Consortium** The RESIST SI Cohort Investigators consist of the persons named in the authors list and the following persons: Berislav Bošnjak^5,11^, Rodrigo Gutierrez Jauregui^5^, Felix Jenniches^4^, Norman Klopp^3^, Till Robin Lesker^8^, Martin Stangel^7^.

The corrected consortium section is shown below.

**Consortium** The RESIST SI Cohort Investigators consist of the persons named in the authors list and the following persons: Berislav Bošnjak, Institute of Immunology, Hannover Medical School and Cluster of Excellence RESIST (EXC 2155) (MHH), Hannover, Germany; Rodrigo Gutierrez Jauregui, Institute of Immunology, Hannover Medical School (MHH), Hannover, Germany; Felix Jenniches, Department for Epidemiology, Helmholtz Centre for Infection Research (HZI), Braunschweig, Germany; Norman Klopp, Hannover Unified Biobank (HUB), Hannover Medical School (MHH), Hannover, Germany; Till Robin Lesker, Department of Microbial Immune Regulation, Helmholtz Centre for Infection Research (HZI), Braunschweig, Germany; Martin Stangel, Department of Neurology, Hannover Medical School (MHH), Hannover, Germany. 

The publisher sincerely apologizes for this error and any inconvenience caused to the authors and readers of the journal.

The original article has been corrected.

