# Supplementary material for: The RESIST Senior Individuals Cohort: Design, participant characteristics and aims
Source: GeroScience. 2024 Aug 14;47(3):3299–310. doi: 10.1007/s11357-024-01299-6 (PMC12181556; doi:10.1007/s11357-024-01299-6)
Supplement: Supplementary file 1 — Supplementary Material 1 [file 11357_2024_1299_MOESM1_ESM.pdf]

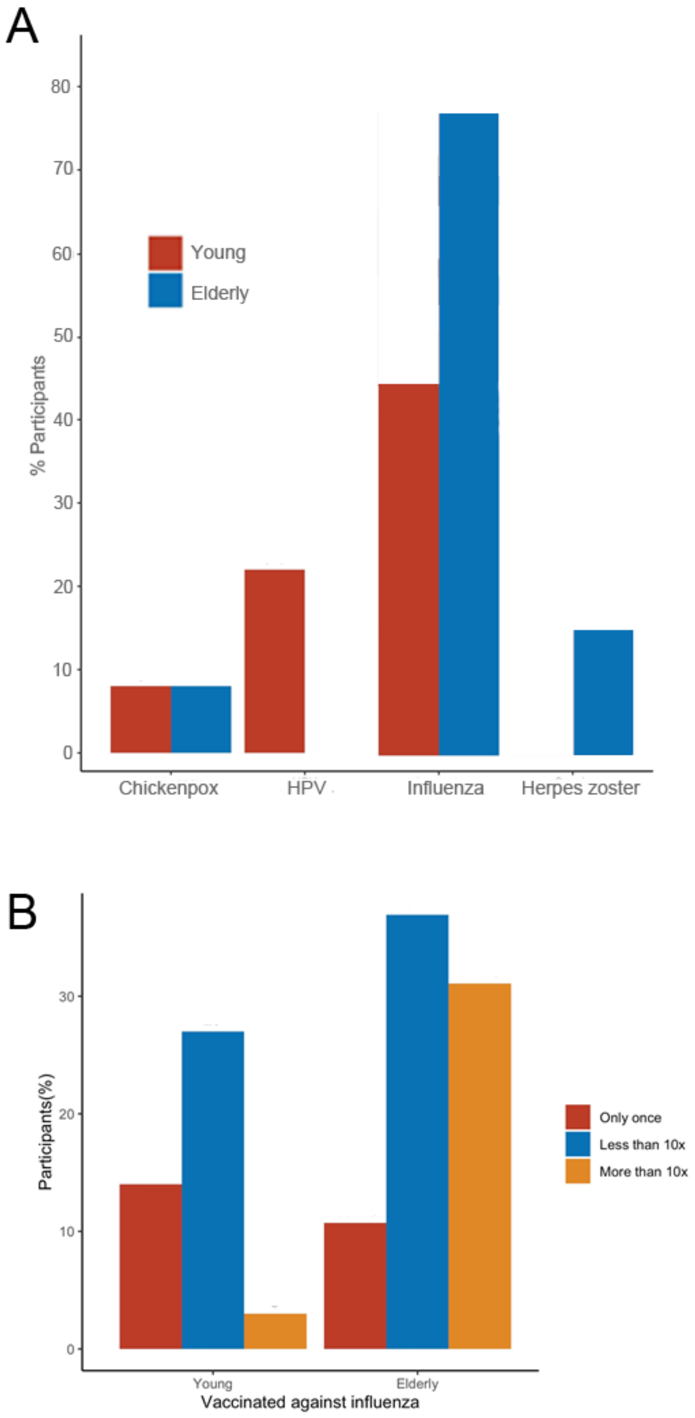

Supplemental Figure 1: (A) Frequency of participant-reported vaccination(s) against Chickenpox, Human papilloma virus, Influenza, and Herpes zoster. (B) Participant-reported vaccination(s) against Influenza in detail.

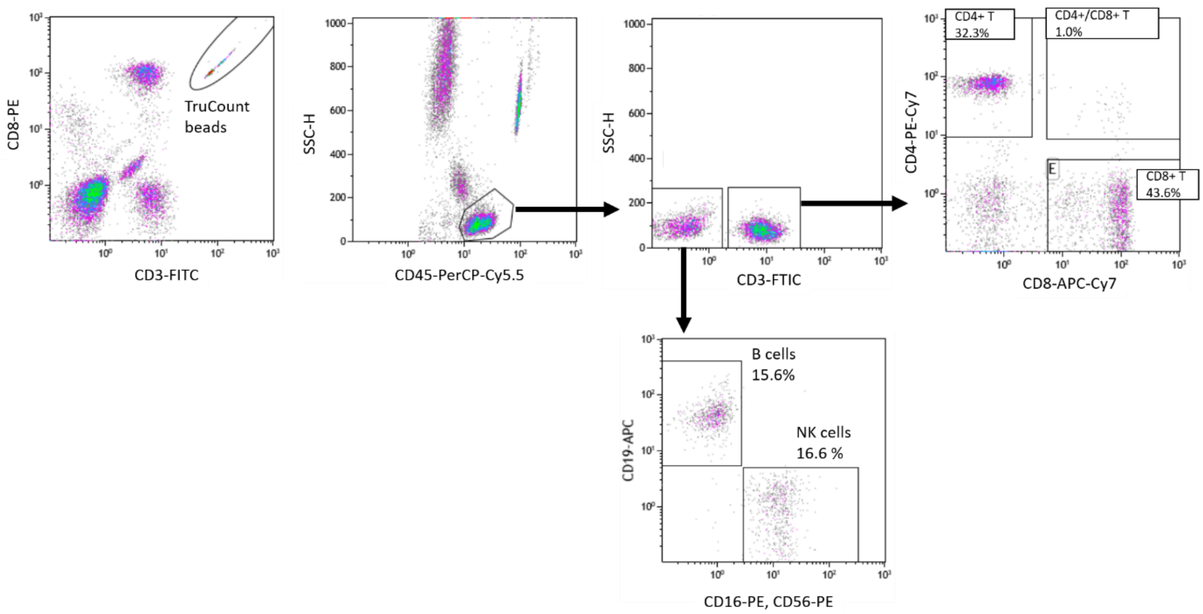

Supplemental Figure 2: Gating strategy of the basic immunophenotyping.
